# Use of a multifocal electroretinogram to evaluate the therapeutic effect of a single intravitreal dexamethasone implant, Ozurdex^®^, for refractory diabetic macular oedema

**DOI:** 10.1186/s40942-025-00652-x

**Published:** 2025-03-19

**Authors:** Aya Refaat Ali Mohammed, Mohamed Farouk Sayed Othman, Yehia Mahmoud Khairat, Amr Ahmed Mohamed Abdelrahman

**Affiliations:** 1 Resident of Ophthalmology, Minia Health Insurance Hospital, Minya, Egypt; 2 Faculty of Medicine, Minia University, Minya, Egypt; 3 Faculty of Medicine, Minia University, Minya, Egypt

**Keywords:** Dexamethasone implant, Refractory diabetic macular oedema, Multifocal electroretinogram, Central retinal thickness, Ozurdex

## Abstract

**Aim:**

To evaluate the therapeutic effect of a single intravitreal dexamethasone implant (Ozurdex^®^) in eyes with refractory diabetic macular oedema (DME) anatomically via optical coherence tomography (OCT) and functionally via best corrected visual acuity (BCVA) and multifocal electroretinography (mfERG).

**Methods:**

This prospective interventional study included twenty eyes with refractory DME that were treated using six intravitreal injections of anti-vascular endothelial growth factor (VEGF). The central retinal thickness (CRT) was measured via OCT exceeding 300 μm. The eyes were treated with a single dexamethasone (DEX) implant four weeks after the last injection of anti-VEGF. The outcomes included changes in CRT, BCVA and p1 amplitude of ring 1 on mfERG and intraocular pressure (IOP) recorded before injection and two, four and six months after DEX injection.

**Results:**

The study included fifteen males (75%) and five females (25%). The mean age was 62.83 ± 6.34 years, with the mean duration of diabetes was 16.7 ± 2.21 years. During the two-month follow-up, there were statistically significant reductions in CRT and logMAR BCVA as well as an increase in p1 of ring 1 on mfERG (*P* = 0.046, *P* < 0.001 and *P* < 0.001, respectively). At four months, these changes were not statistically significant (*P* = 0.99, *P* < 0.56&*P* < 0.58), whereas at six months, all the parameters nearly reached pre-DEX injection values (*p* = 0.93 *P* = 0.99 *P* = 0.81). The IOP values were not significantly increased at two, four or six months (*p* < 0.06, *P* = 0.35 and *P* = 1.0, respectively). There were significant negative correlations between the mfERG and OCT parameters before and six months after DEX injection (*p* = 0.000).

**Conclusion:**

A single intravitreal injection of DEX in refractory DME patients induced significant anatomical and functional improvements, but these improvements only lasted for short periods of up to four months. This treatment exhibited an excellent safety profile. However, at six months, the therapeutic effect was null. The utility of mfERG as a sensitive biomarker of treatment efficacy was highlighted herein.

## Introduction

DME is the main cause of visual compromise in diabetic patients [1]. DME is considered refractory or persistent when the patients are treated monthly by intravitreal anti-VEGF for at least six injections and have a CRT exceeding 250 μm with associated visual loss [2]. Protocol T indicates that better treatment outcomes would be obtained by increasing the loading dose of intravitreal injection of anti-VEGF for DME to five injections, particularly with aflibercept [3]. Chronic inflammation is the major pathogenic pathway in refractory DME [4]. There are several inflammatory mediators involved in the pathogenesis of refractory DME, such as VEGF, monocyte chemoattractant protein-1, tumour necrosis factor-alpha, interleukin-6, interleukin-8, and cyclooxygenase-2 [5, 6]. Corticosteroids have an anti-inflammatory effect and thus may be considered as an alternative treatment for refractory DME [1, 7]. Ozurdex^®^ is an intravitreal implant consisting of micronized dexamethasone in a biodegradable copolymer of polylactic-co-glycolic acid. It slowly releases steroids into the vitreous over approximately 6 months [8]. The United States Food and Drug Administration (FDA) and most European countries approved the use of DEX to treat DME in 2014 [9].

OCT reveals several morphological changes that could affect the treatment outcome of DEX implants for DME [10]. Disorganization of the retinal inner layers (DRILs) is one of these changes [11]. Additionally, this imaging approach can be used to identify biomarkers for plana vitrectomy and peeling of the internal limiting membrane in naïve DME [12]. Ultra-wide-field scanning laser ophthalmoscopy is another imaging modality for diabetic retinopathy (DR) [13]. The association between retinal structural and functional alterations in DME patients has rarely been thoroughly investigated using OCT and mfERG together [14].

## Methods

### Study subjects and design

This prospective interventional study included twenty eyes from twenty patients with refractory DME. The patients were recruited from the Ophthalmology Departments of Minia Health Insurance Hospital and Faculty of Medicine Minia University between December 2022 and February 2024. The study was approved by the Faculty of Medicine at Minia University through Medicine’s Institutional Review Board (Approval number: 515–2022). All patients provided informed written consent for their study participation.

The inclusion criteria were as follows: pseudophakic patients with an intact posterior capsule; patients with DME who received six monthly injections of anti-VEGF (three ranibizumab agents followed by three aflibercept agents); and patients who had a CRT exceeding 300 μm after receiving Ozurdex^®^ at least 4 weeks after the last anti-VEGF injection.

Patients with a previous history of pars plana vitrectomy, ocular surgery in the last six months, laser retinal photocoagulation, ruptured posterior capsule, aphakia, glaucoma, poor fixation, macular ischaemia on fluorescein angiography, proliferative diabetic retinopathy and concomitant other ocular pathologies that may affect macular function or visual acuity were excluded from the study.

Full general and ophthalmological evaluations were performed, including a detailed medical history, a BCVA assessment via the Snellen chart (measurements were converted to logarithms of the minimum angle of resolution (log-MAR) equivalents for data analysis), IOP measurement via Goldmann applanation tonometry, slit-lamp examination of the anterior segment, fundus biomicroscopy via a 78D lens and binocular indirect ophthalmoscopy.

### Ophthalmologic imaging

#### Optical coherence tomography (OCT)

Spectral-domain optical coherence tomography (SD-OCT) was performed (Topcon 3D OCT-2000, Tokyo). Two scanning protocols (Retina Map and Radial Scans) were selected for each patient. The central macular thickness in the central 500 μm centred on the fovea, which corresponds to central ring 1 of mfERG, was measured.

#### Multifocal electroretinogram (mfERG)

**Fig. 1:**
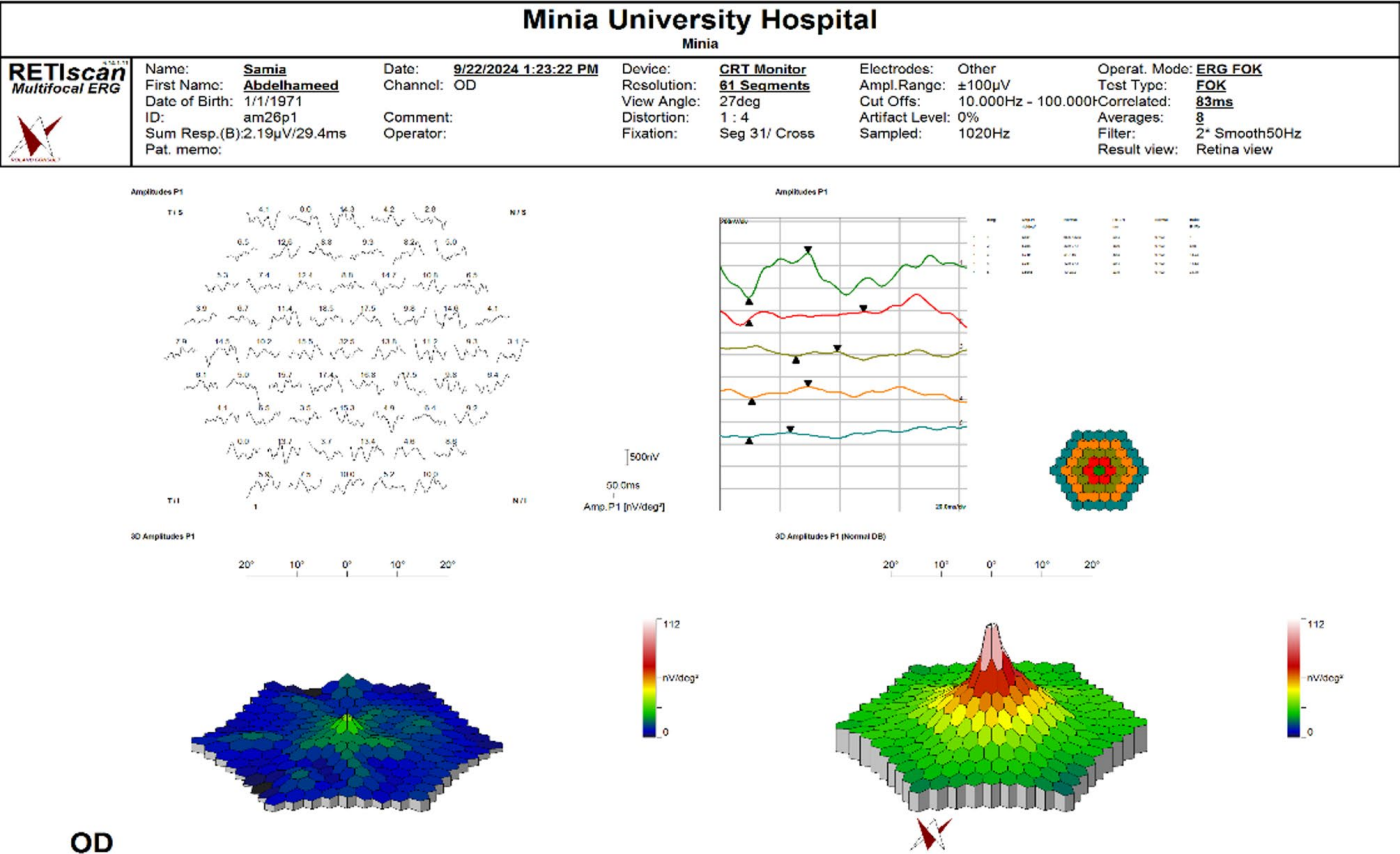
Showed mfERG displays with array display at top left, ring display at top right and 3D display of the patient at bottom left and the standard 3D display at bottom right

mfERG was performed via the RETI-Port/Scan 21 platform (Roland Consult, Wiesbaden, Germany). The active electrode used was an HK-Loop, which consists of thin stranded monofilaments applied to the lower fornix. The amplitude densities of the initial positive peak (P1) of ring 1 measured in nanovolts per degree squared (nV/deg2). The print of mfERG included an array display at the top left, a ring display at the top right, a 3D display of the patient at the bottom left and a standard 3D display at the bottom right, as shown in Fig. 1. Our goal in this study was to explore the ring display and examine its correlation with CRT measured by OCT.

#### Fluorescein angiography (FA)

Fluorescein angiography (FA) was performed for diabetic patients via a Zeiss Visucam 500 (Carl Zeiss Meditec AG, Inc., Jena, Germany) to rule out macular ischaemia.

#### Intravitreal dexamethasone implant injection

**Fig. 2:**
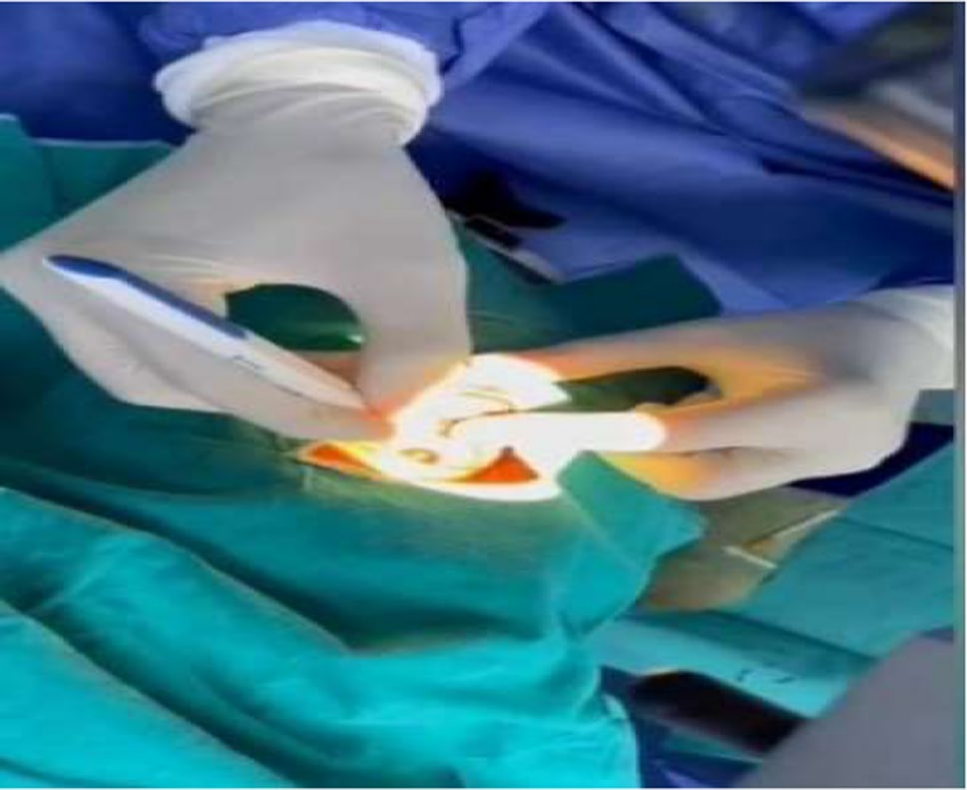
Injection of Ozurdex^®^ 700 µg was through the pars plana using its specific injector systems in sterile operating room

**Fig. 3:**
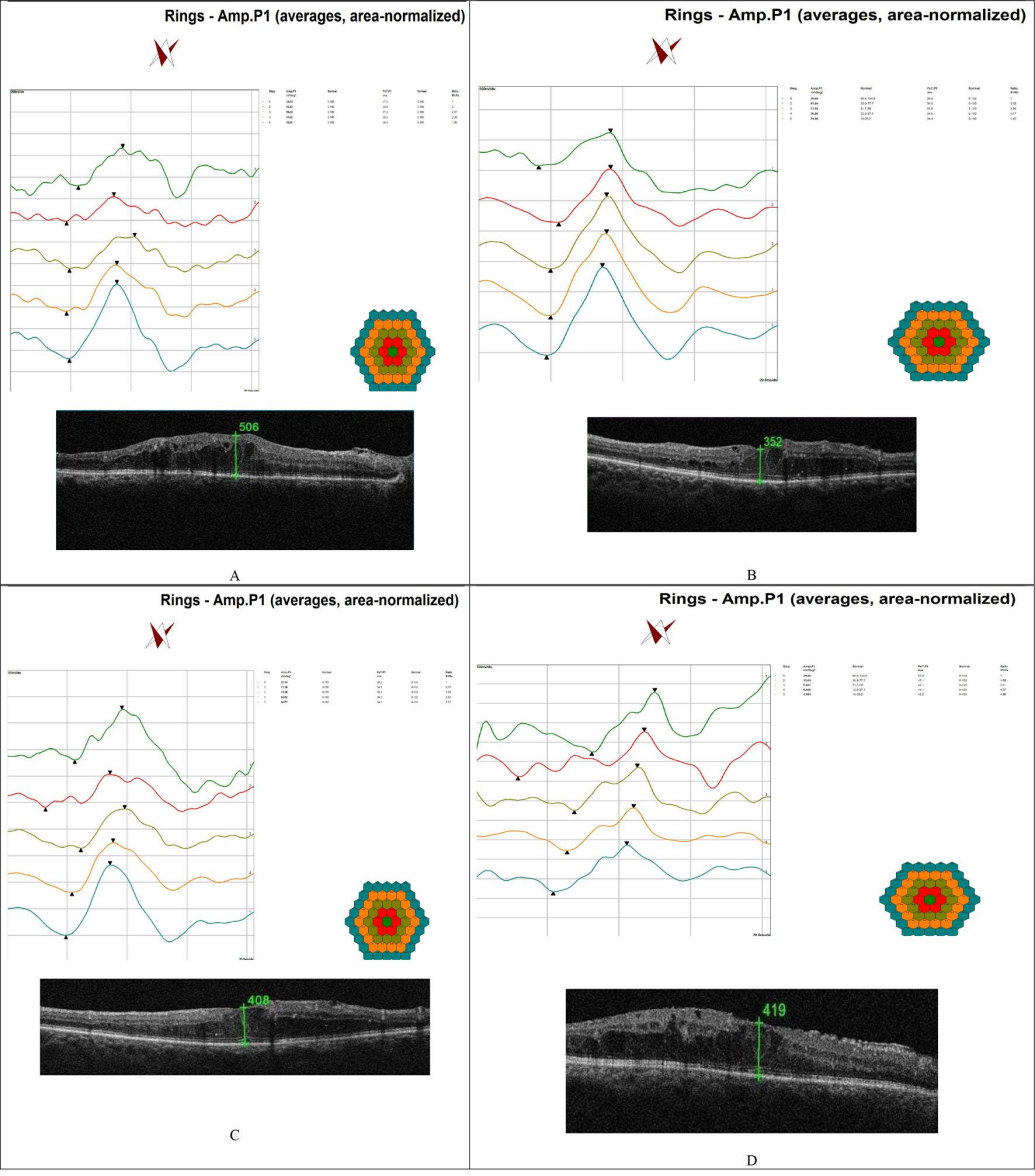
Ring display of mfERG and CRT measured by OCT pre-DEX injection and post DEX injection at two, four and six months follow up periods. **A**: showed pre-DEX injection values of p1 amplitude of ring 1 measure 24.93 nV/deg2 and CRT 506 μm. **B**: same patient two months post DEX injection showed p1 amplitude of ring 1increased to 36.05 nV/deg2 and CRT decreased to 352 μm. **C**: same patient four months post DEX injection showed p1 amplitude of ring 1 measure 27.14 nV/deg2 and CRT 408 μm. **D**: same patient six months post DEX injection showed p1 amplitude of ring 1 measure 24.56 nV/deg2 and CRT 419 μm

A 700-µg dose of dexamethasone (Ozurdex^®^, Allergan Inc., Irvine, CA, USA) was injected through the pars plana via a specific injector system under topical anaesthesia in a sterile operating room. After sterilization, the skin and conjunctiva were sterilized with 10% and 5% povidone-iodine, respectively, as shown in Fig. 2. After injection, a topical antibiotic was applied four times per day for one week Fig. 3.

### Statistical analysis

The data were statistically analysed via the Statistical Package for Social Sciences (SPSS) program (software version 25; SPSS Inc., IBM Corp., New York, USA, 2017).

Normally distributed quantitative data are expressed as means, standard deviations (SDs), and ranges. A t test was used to test the significance of differences between two groups. Nonnormally distributed data were compared via the Mann‒Whitney U test. Pearson’s correlation analysis was performed to examine associations between normally distributed variables. A P value less than 0.05 was considered to indicate statistical significance (P value ≤ 0.05).

## Results

### Demographic and clinical features

Twenty eyes of twenty patients with refractory DME were included in the study.

The ages of the patients ranged from 52 to 73 years, with a mean of 62.83 ± 6.34 years. There were 15 (75%) males and 5 (25%) females. The duration of diabetes ranged from 13 to 21 years, with a mean of 16.7±2.21 years. The CRT (measured via OCT), logMAR BCVA, P1 amplitude of central ring 1 oonf mfERG and IOP were recorded for each patient at 2 months, 4 months and 6 months after DEX implantation and compared to pre-DEX injection values.

With respect to CRT, there was a statistically significant reduction at two months (470.7 ± 126.6 to 333.2 ± 87.3, p1 = 0.046*). However, this reduction was no longer significant at four months (470.7 ± 126.6 to 457.5 ± 201.08 *p* = 0.99), and at 6 months, the CRT nearly reached the preinjection value (470.7 ± 126.6 to 472.8 ± 256.4 *p* = 0.93) (Table 1).

**Table 1:** Comparison of CRT, P1 amplitude, IOP, and log-MAR BCVA at different time intervals post DEX injection to pre-DEX injection values

	Pre-DEX	2 m post DEX	4 m post DEX	6 m post DEX
CRT	470.7 ± 126.6	333.2 ± 87.3	457.5 ± 201.08	472.8 ± 256.4
P value with pre injection		p1 = 0.046.	*P* = 0.99	*P* = 0.93
P1 amplitude of mfERG	24.79 ± 13.54	37 ± 2.1	30.96 ± 13.3	25 ± 2.2
P value with pre injection		*P* < 0.001	*P* = 0.58	*P* = 0.81
IOP	19.11 ± 3.4	21 ± 3.32	20 ± 3.31	19.3 ± 3.29
P value with pre injection		*P* = 0.06	*P*= 0.35	*P* = 1.0
log-MAR BCVA	1.08 ± 0.27	0.71 ± 0.28	0.96 ± 0.28	1.06 ± 0.33
P value with pre injection		*P* < 0.001	*P* = 0.56	*P* = 0.99

With respect to the P1 amplitude of ring 1, there was a statistically significant increase at 2 months (24.79 ± 13.54 to 37 ± 2.1 *p* < 0.001). At 4 months, this increase was no longer statistically significant (24.79 ± 13.54 to 30.96 ± 13.3 *P* = 0.58), and at 6 months, the P1 amplitude of ring 1nearly reached the baseline value (24.79 ± 13.54 to 25 ± 2.2 *P* = 0.81) (Table 1).

The logMAR BCVA exhibited a significant decrease at 2 months (1.08 ± 0.27 to 0.71 ± 0.28, *P* < 0.001), but the changes were nonsignificant at 4 months (1.08 ± 0.27 to 0.96 ± 0.28, *P* = 0.56) and 6 months (1.08 ± 0.27 to 1.06 ± 0.33, *P* = 0.99) (Table 1).

The IOP values did not exhibit a significant increase at 2 months (19.11 ± 3.4 to 21 ± 3.32 *p* < 0.06), 4 months, (19.11 ± 3.4 to 20 ± 3.31 *P* = 0.35) or 6 months (19.11 ± 3.4 to 19.3 ± 3.29 *P* = 1.0), thus indicating that there were no significant IOP fluctuations (Table 1).

There were significant negative correlations between CRT and P1 amplitude at baseline and at six months after DEX injection (*r* = -0.949, *p* = 0.000* and *r* = -0.901, *p* = 0.000*, respectively; Table 2).

**Table 2:** Correlation betweenP1 amplitude of MfERG and CRT pre and post DEX injection at different interval times

		MFERG pre injection	MF after 1 M	MF after 2 M	MF after 6 M
OCT pre injection	R	-0.949	-0.087	-0.250	-0.906
	P	0.000	0.714	0.288	0.003
OCT after 1 M	R	-0.165	0.203	0.121	-0.148
	P	0.488	0.391	0.611	0.533
OCT after 2 M	R	-0.393	0.248	0.093	-0.326
	P	0.087	0.291	0.698	0.161
OCT after 6 M	R	-0.895	0.256	0.093	-0.901
	P	0.000	0.275	0.793	0.000

## Discussion

The role of chronic inflammation in DME has been examined by several studies [15–18]. Previous studies reported a short-term effect of DEX injection, peaking at 1–3 months and starting to deteriorate at 4–6 months, thus requiring repeated injection after 3 months [19]. Therefore, the follow-up recordings schedule of patients included in this study were at 2 months, 4 months and 6 months post DEX implant; this follow-up regimen was consistent with that of Castro-Navarro V et al. [20]. The same SD-OCT device was used for all scans to avoid systematic bias that could occur with use of different OCT modalities [21]. In this study, there was no significant increase in IOP at any of the follow-up assessment. This finding was consistent with the results reported by Nalcaci et al., who found no significant change in IOP during the 6 months follow-up period [22]. This findings indicated the safety of single injection of intravitreal DEX with respect to changes in IOP. However, the IOP values obtained herein were lower than those reported in other studies such as the MEAD, BEVORDEX, and RELDEX trials. This difference is due to the number of injections, since it has been shown that increases in IOP are greater with repeated DEX injections [23–26]. Long-term therapeutic effect over 24 months of follow-up with repeated DEX injections for patient with DME who were treatment-naïve and refractory to anti-VEGF induced improvements in BCVA and reductions in CST [27].Additionally, this treatment approach has the potential to not only delay progression of DR but may also improve DR severity [28]. This study revealed a statistically significant decrease in log-MAR BCVA and a statistically significant reduction of CRT (measured by OCT) in the short term (2 months after DEX injection), consistent with findings from previous studies that evaluated the therapeutic effect of single DEX implant for refractory DME [22, 29–32]. These results indicate that early beneficial functional and anatomical improvements can be induced by a single intravitreal DEX implant for refractory DME. At the 4-month follow-up, the therapeutic effects of DEX weakened gradually, and changes of BCVA and CRT were no longer statistically significant; at 6 months, these values nearly reached their baseline values. Previous reports evaluated a single intravitreal DEX implant for refractory DME did not exhibit treatment effects at six months [29, 31, 32]. The weakening of the therapeutic effect at 6 months post DEX implant in refractory DME is consistent with the findings of Zarranz-Ventura, Javier, et al. [33]. Additionally, a previous study conducted by Gutierrez-Benitez, L., et al. with mean follow-up period of 7.6 months found that further treatment with intravitreal DEX was required in 43% of the patients [30]. However, the findings of our study were inconsistent with those of Nalcaci et al., who concluded that intravitreal DEX injection is associated with significant reductions in CRT up to six months after treatment. This difference can be attributed to variations in the inclusion criteria; Nalcaci et al. included patients who were resistant to at least 3 monthly ranibizumab injections, while the current study included patients resistant to six injections of anti-VEGF [22]. This study evaluated the functional treatment effect of DEX implant on BCVA as well as on the p1 amplitude of ring 1 on mfERG. There was a significant increase in the p1 amplitude of ring 1 at two months after DEX injection; however, the increase at four months was no longer significant, and the amplitude nearly reached its preinjection value at six months. These results were consistent with those of Karacorlu, M., et al., who documented statistically significant increases in the mean P1 response amplitude at one and three months post intravitreal injection; however, they evaluated the therapeutic effect of intravitreal triamcinolone acetonide on DME [34]. These results are inconsistent with those of Mastropasqua et al., who found that DEX causes only stability of retinal function at 4 months after injection; retinal function becomes worse after that period [35]. This different outcome results on mfERG at 4 months due to other structural OCT abnormalities other than CRT that could affect macular function such as disorganization of retinal inner layers (DRIL) and intraretinal cysts [14]. Additionally, the results of this study are inconsistent with the findings of Tranos et al., who found a significant increase in the p1 amplitude of ring 1 on mfERG at six months after DEX injection for DME. This difference can be attributed to the performance of mfERG at baseline and at six months. DEX implants were readministered on a PRN regimen to twenty eyes from twenty eight patients eyes included in the study; therefore, the significant increase at six months was due to repeated implants. Additionally, the study design was different, as Tranos et al. included both treatment-naïve eyes and refractory DME patients, while this study included refractory patients only [36].

This study’s limitations include a relatively small number of eyes and a single-center design, which may limit the strength of data analyses. The lack of a control group is a major drawback, as it is essential to differentiate whether the results observed in the study are due to the natural progression of the DME or the effects of DEX implants. Additionally, short follow up period of the study makes it can’t conclude long term efficacy and treatment frequency.

## Data Availability

No datasets were generated or analysed during the current study.
